# Commentary: The mutations and clinical variability in maternally inherited diabetes and deafness: an analysis of 161 patients

**DOI:** 10.3389/fendo.2023.1205862

**Published:** 2023-06-07

**Authors:** Ivo P. van de Peppel, Serwet Demirdas, Behiye Özcan

**Affiliations:** ^1^Department of Clinical Genetics, Erasmus Medical Centre, Rotterdam, Netherlands; ^2^Department of Clinical Genetics, Leiden University Medical Centre, Leiden, Netherlands; ^3^Department of Internal Medicine, Erasmus Medical Centre, Rotterdam, Netherlands

**Keywords:** MIDD, heteroplasmy, mitochondrial disease, diabetes mellitus, m.3243A>G, clinical genetics

## Introduction

Yang and colleagues reviewed the current, but still scarce, literature on maternally inherited diabetes and deafness (MIDD) (1). The study clearly describes patient characteristics, and raises clinical awareness of signs that could help distinguish MIDD from other types of diabetes. However, the authors also establish a correlation between the levels of heteroplasmy seen in the causal genetic variants and clinical phenotype. In this commentary we would like to place a cautionary note to this finding, and emphasize the heterogeneity in clinical presentation of MIDD and the difficulty of using heteroplasmy levels to predict the clinical phenotype.

## The heterogeneity in clinical presentation of genetic variants leading to MIDD

MIDD is most commonly caused by the m.3243A>G variant in mitochondrial DNA (mtDNA). When present in high heteroplasmy levels in the brain (>70%), this variant can also lead to a condition called mitochondrial encephalomyopathy, lactate acidosis and stroke-like episodes (MELAS) (2). The m.3243A>G variant was present in 82% of the patients with MIDD with clinical diabetes analyzed by Yang et al. (n=136). A wide variation in clinical presentation was observed: apart from diabetes, most MIDD patients displayed hearing loss (85.7%). Central nervous system issues, myopathy, heart problems, polyneuropathy and eye disease, were also relatively common (17-30%). A recent study suggests clinical symptoms are even more heterogeneous in m.3243A>G variant carriers (3). A study from 2012 that was not included in the review by Yang et al. analyzed 72 carriers of the m.3243A>G variant (from 34 families) (4). In these individuals, hearing loss was reported as the most prevalent symptom (48%). Glucose intolerance was only reported as the fifth most common clinical expression (38%). Gastro-intestinal symptoms were reported as the second most common sign (42%). This contrasts with the result of Yang et al. who reported a prevalence of gastro-intestinal symptoms of just 5.6%. Methods of data collection and availability of patient characteristics vary between studies potentially leading to under or over-reporting certain clinical signs. One big difference between the studies is that De Laat et al. included all variant carriers while Yang et al. included variant carriers with clinical diabetes. However, these differences still underline the heterogeneity of MIDD-MELAS disease spectrum of which clinicians should be aware.

## Diagnosis of MIDD patients using blood and urine heteroplasmy levels

MIDD is diagnosed by analyzing mtDNA that can be isolated from various cell types. Leukocytes from blood are most commonly used because of easy accessibility. However, heteroplasmy levels in blood go down with age and might poorly reflect heteroplasmy in other cell types (5–7). The reason for the differences in heteroplasmy between cell types and for the age-related decline is incompletely understood. Suggested explanations include genetic drift or selection against a particular variant (7). Because of this variation in heteroplasmy levels across tissues and age groups, many intra- and inter-individual outliers are seen (4, 5). In older patients it is more common to observe low blood and concurrently high urine heteroplasmy levels. Unsurprisingly, attempts to correlate heteroplasmy levels with various clinical metrics, such as age-of-onset of diabetes, have produced conflicting results. For example, Yang et al. describe a significant negative correlation between blood heteroplasmy levels and age of onset of diabetes, while a recent study by Sakata et al. found no such correlation, even when correcting for age (8). Sakata et al. only observed a correlation between heteroplasmy levels and age of onset of hearing loss. Due to the variability of heteroplasmy levels it is therefore advisable to test both blood and another cell type to diagnose MIDD [e.g. through muscle biopsy or urine (9)]. In older patients suspected of MIDD, one might consider testing urine directly because blood is less reliable. Lastly, due to the variability of heteroplasmy levels, clinicians should be wary of using them to predict clinical outcomes.

## Discussion

The heterogeneity in clinical phenotype of the MIDD-MELAS disease spectrum is frequently attributed to heteroplasmy levels varying in different tissues (e.g. high levels in the brain result in MELAS and high levels in the pancreas in diabetes mellitus) (5, 7). Studies on heteroplasmy levels in brain tissue of symptomatic MELAS patients of both children and adults quite consistently display levels of >70% (2). Data on heteroplasmy levels in other affected tissues in MELAS or MIDD patients is limited. Older post-mortem studies of MELAS patients (aged between 12 and 23) have demonstrated that heteroplasmy levels are generally around 60 to 80% in all measured tissues (including pancreas, liver, myocardium, intestine, and kidney) (10, 11). Whether these tissue-specific heteroplasmy levels correspond to the severity of a specific phenotype (e.g. pancreatic heteroplasmy levels and clinical diabetes) has not been directly assessed for MIDD. In clinical practice it is both unfeasible and undesirable to test heteroplasmy levels in every tissue of a patient. Heteroplasmy levels in the blood and preferably in urine and/or muscle are of great importance as a diagnostic tool for MIDD (9). However, due to the high degree of variability and its decline with age, correlating blood heteroplasmy levels to clinical outcomes as Yang et al. (and others) have done, requires a high degree of caution (1, 8). It is possible that at a cohort level in a research setting, with corrections for potential confounders like age, such correlations could provide interesting insights. However, we advise against using heteroplasmy levels in clinical practice to predict outcome in individual patient cases. It is likely that the severity of the and progression of the MIDD phenotype is affected by many other factors including sex, environmental, and genetic modifiers [recently reviewed in-depth by (3)]. When an MIDD variant is discovered, all patients should undergo a complete clinical work-up to assess potential symptoms in different organ systems (12). Future research is required to create a more elaborate model to potentially help predict the severity and progression of the MIDD phenotype in clinical practice. This will likely include other variables, such as heteroplasmy levels in more than one tissue, age, and other environmental and genetic modifiers.

## Author contributions

IP, SD and BO contributed to the conception of the commentary. IP wrote the manuscript. IP, SD and BO read, reviewed, revised and approved the submitted version.
